# Retraction Note: Gut Microbiota Alteration is Characterized by a Proteobacteria and Fusobacteria Bloom in Kwashiorkor and a Bacteroidetes Paucity in Marasmus

**DOI:** 10.1038/s41598-023-44593-7

**Published:** 2023-10-30

**Authors:** Thi-Phuong-Thao Pham, Maryam Tidjani Alou, Dipankar Bachar, Anthony Levasseur, Souleymane Brah, Daouda Alhousseini, Cheikh Sokhna, Aldiouma Diallo, Frank Wieringa, Matthieu Million, Didier Raoult

**Affiliations:** 1 Aix-Marseille Univ, IRD, APHM, MEPHI, IHU-Méditerranée Infection, Marseille, France; 2 Institut de cancérologie Gustave Roussy Cancer Campus, Villejuif, France; 3 Service de Médecine Interne et Générale, Hôpital de Niamey, Niamey, Niger; 4 Unité de Recherche sur les Maladies Infectieuses et Tropicales Emergentes IRD 198, Centre National de la Recherche Scientifique 7278, Aix-Marseille Université, Dakar, Senegal; 5 Institut de Recherche pour le Développement (IRD) - IRD/UM/SupAgro, Montpellier, France

Retraction of: *Scientific Reports* https://doi.org/10.1038/s41598-019-45611-3, published online 24 June 2019

Editors have retracted this Article.

After publication of this paper concerns about ethical oversight of this study were brought to the attention of the Editors. The Authors were not able to provide documentation of appropriate approval from an ethics committee in either Niger or Senegal, where the participants in this study were based.

Thi-Phuong-Thao Pham, Cheikh Sokhna, Frank Wieringa, Mathieu Million, and Didier Raoult disagree with this retraction. Maryam Tidjani Alou, Dipankar Bachar, Anthony Levasseur, Souleymane Brah, and Daouda Alhousseini did not respond to correspondence from the Editors about this retraction. The Editors were not able to confirm current contact details for Aldiouma Diallo.

